# Effects of different proteins and maltodextrin combinations as wall material on the characteristics of *Cornus officinalis* flavonoids microcapsules

**DOI:** 10.3389/fnut.2022.1007863

**Published:** 2022-09-14

**Authors:** Mengyue Zhao, Weiwei Cao, Linlin Li, Aiqing Ren, Yuan Ang, Junliang Chen, Bhesh Bhandari, Zhe Wang, Xing Ren, Guangyue Ren, Xu Duan

**Affiliations:** ^1^Department of Food Sciences and Engineering, College of Food and Bioengineering, Henan University of Science and Technology, Luoyang, China; ^2^Department of Food Science, Institute of Food Research, Hezhou University, Hezhou, China; ^3^Department of ARC Dairy Innovation Hub, School of Agriculture and Food Sciences, The University of Queensland, Brisbane, QLD, Australia

**Keywords:** *Cornus officinalis* flavonoids, microcapsules, protein, maltodextrin, quality

## Abstract

The flavonoids in *Cornus officinalis* (CO) have various pharmacological activities, however, the flavonoid instability limits its application in food and pharmaceutical industries. In this study, *Cornus officinalis* flavonoid (COF) microcapsules were prepared by using a combination of whey isolate protein (WPI), soy isolate protein (SPI), gelatin (GE), and maltodextrin (MD) as wall materials, respectively. Meanwhile, the encapsulation efficiency, solubility, color, particle size, thermal stability and microstructure as well as the antioxidant capacity of microcapsules were assessed. When the protein/MD ratio was 3:7, three kinds of combined wall materials realized high encapsulation efficiency (96.32–98.24%) and water solubility index (89.20–90.10%). Compared with other wall material combinations, the microcapsules with WPI-MD wall ratio at 3:7 had lower particle size (7.17 μm), lower moisture content (6.13%), higher encapsulation efficiency (98.24%), better water solubility index (90.1%), higher thermal stability (86.00°C), brightness L^*^ (67.84) and higher 1,1-diphenyl-2-picrylhydrazyl (DPPH) scavenging capacity (6.98 mgVc/g), and better flowability. Results suggested that WPI and MD could be better wall materials applied in encapsulating COF.

## Graphical Abstract

Schematic description about the microencapsulation of *Cornus officinalis* flavonoid extract by freeze-drying technology. 
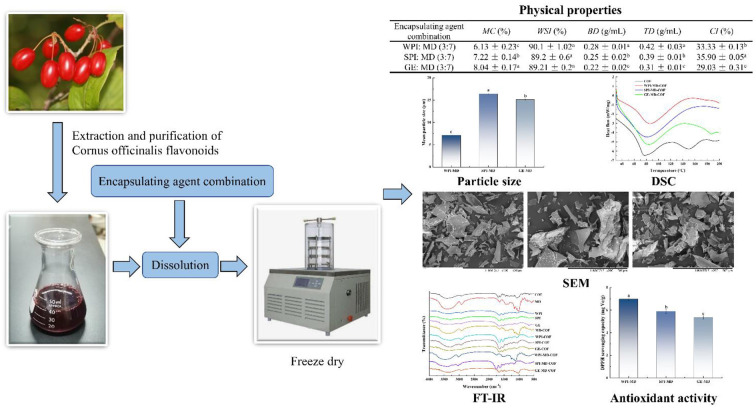

## Introduction

*Cornus officinalis* (CO) is known as the genus *Cornus* L, belonging to the Cornaceae family, which is a kind of traditional Chinese medicine and food homology (1). CO is mainly distributed in China, South Korea, and Japan (2). CO tastes sour and astringent, and is often used to improve liver and kidney function. Many studies have shown that CO is rich in flavonoids, pentacyclic triterpenoids, iridoid glycosides, and other compounds, which is often used for nourishing the liver and kidneys (3). Flavonoids are the second main components of CO (221.3 mg/10 g) (4), which can be divided into quercetin, kaempferol, rutin, and naringin according to their structure types. Modern pharmaceutical studies have proved that flavonoids have various biological activities such as antioxidant (5), antibacterial (1), hypoglycemic (6), and antiatherosclerosis (7) activities. However, flavonoids are sensitive to light, heat, and food processing conditions, so the application of *Cornus officinalis* flavonoids (COF) in food industry is limited (8). Simulated human gastrointestinal digestion studies demonstrated COF has poor stability in the gastrointestinal tract and is easily degraded. Intestinal digestion resulted in a substantial decrease in the content and antioxidant activity of the fruit extracts, which limits its physiological activity *in vivo* (9). Therefore, it is crucial to protect COF from unfavorable conditions.

Microencapsulation is a common technology to encapsulate bioactive compounds into microcapsules or nanoparticles, which can prevent the encapsulated substance from contacting external environment, effectively protect biological activity, improve storage stability, and conceal the bitter taste (10). Various studies on the methods of encapsulating bioactive compounds have been studied, including spray drying (SD), spray freeze drying, freeze drying (FD), complex coalescence, and electrospraying (11). Among them, FD could make microcapsules dried by sublimation under high vacuum conditions and lower temperature. FD is deemed a suitable method for encapsulating bioactive compounds sensitive to heat and oxygen (11, 12). At present, natural biopolymers commonly used for embedding flavonoid compounds contain starch, maltodextrin, acacia gum, whey protein isolate, soy protein isolate, gelatin, and protein-polysaccharide combination (13). However, encapsulation products prepared by different coated wall materials have different physicochemical properties. Although a variety of natural biological polymers used for encapsulating flavonoids have been reported, microcapsules fabricated with single coating wall always has several limitations such as low antioxidant capacity (14), poor encapsulation efficiency (15), and unsatisfactory solubility. However, microcapsules prepared by protein-polysaccharide combination as wall materials show many advantages on encapsulation efficiency (EE) and stability. For example, the EE of curcumin encapsulated by coconut whey powder and gum Arabic was higher than that of curcumin encapsulated by single coconut whey powder (16); Hu et al. (17) showed that citrus flavone microcapsules prepared by whey protein concentrate and acacia gum complex had better retention efficiency after 3 months storage than that prepared by single acacia gum. Previous studies reported that the EE of cornelian cherry (*Cornus mas* L.) polyphenols encapsulated by β-cyclodextrin was 65.62% (18), however, protein-polysaccharide combination as better wall material to enhance the EE and stability of COF is scarcely reported.

Maltodextrin (MD) is a type of hydrolyzed starch with good solubility and low viscosity. Whey protein isolate (WPI) is a good carrier of bioactive substances because of its good emulsification, gelling, and thickening (19). SPI is a type of gel type, which is easily biodegradable and in high nutritional value. Gelatin (GE) is a hydrolyzed protein with good biocompatibility and high stability (14). The combined wall materials can greatly improve the physical properties of microcapsule products. However, the study on COF microcapsules prepared by MD and the above different protein combination has not been reported.

In this study, the combination of different proteins (WPI, SPI, GE) and MD were used to encapsulate COF. The effects of different combinations on the EE, particle size, thermodynamic behavior, structure, and microstructure of microcapsule products were investigated. This study is aimed to screen the best combination of protein and maltodextrin as wall material for encapsulating COF and expand its application in functional food industry.

## Materials and methods

### Materials

*Cornus officinalis* was purchased from Xixia County, Henan Province. CO was dried using a microwave freeze dryer (MFD) developed by Lujie et al. (20). Fresh Cornus fruit was frozen at −25°C for at least 8 h. The drying chamber pressure was set at −100 Pa, and cold trap temperature was −40°C. The power of microwave was set at 1 W/g. During the drying process, the temperature of the material was detected by an infrared thermal imager. Drying lasted until the dry basis moisture content of the material reached 0.09 g/g dry weight. The dried material was crushed and passed through a 60-mesh sieve and stored at −25°C.

Maltodextrin, rutin and 1,1-diphenyl-2-picrylhydrazyl (DPPH) were purchased from Shanghai Yuan ye Biological Co., Ltd., China. SPI was purchased from Dezhou Gu Shen Protein Technology Biology Co., Ltd., in Dezhou, China. WPI was purchased from Hilmar Corporation, California, USA. GE was purchased from Henan Bo yang Biotechnology Technology Co., Ltd., in Zhou Kou, China. The other reagents were of analytical grade.

### Ultrasonic extraction of COF

*Cornus officinalis* flavonoids was extracted by using an ultrasonic machine according to Wu et al. (21) with slight modification. CO powder (100 g) was added with 70% ethanol (1:15, w/v) and sonicated 3 times at 50°C with ultrasonic power of 300 W for 30 min each time. The filtrate was mixed and centrifuged, and the supernatant was concentrated by a rotary evaporator, and then stored at 4°C.

Flavonoids were purified by the method that Ismail et al. (22) reported with appropriate modifications. First, AB-8 resin was soaked in anhydrous ethanol for 24 h, and the column was wet-loaded. The elution process continued until the effluent was free of white turbidity, and AB-8 resin was washed with distilled water for 1 h. After decompression concentration, the extract was diluted with the appropriate amount of water and then eluted repeatedly with distilled water to remove impurities such as protein and sugar. Then the flavonoids were eluted with ethanol solution (40%), and the eluent was concentrated and freeze-dried to obtain COF.

### Determination of COF

The aluminum chloride method was used to determine the content of total flavonoids (23). The diluted flavonoid extract (1 ml) was mixed with 4 ml 70% ethanol solution in a 10 ml volumetric flask, the reagents of 0.3 ml 5% NaNO_2_ solution and 0.3 ml 10% Al (NO_3_)_3_ solution were further added and shaken for 6 min, and finally 2 ml 4% NaOH solution was added. After 15 min, the absorbance was measured at 510 nm. The content of flavonoids (mg/g) in the extract was calculated according to rutin standard curve.

### Preparation and characterization of microcapsules

WPI, SPI, GE, and MD powders were dispersed in distilled water at different ratios of 2:3, 3:7 and 1:3 (w/w) at 40°C and the mixed carrier solution concentration of 10% (w/w) was obtained. The solution was stirred at 350 ± 25 rpm/min for 2 h. The core material solution was added at a core-wall ratio of 1:9 (w/w) and stirred for 10 min. A high-pressure homogenizer (A25, Shanghai OUHOR Mechanical Equipment Co., Ltd., Shanghai, China) was used for homogenization at 10,000 rpm/min for 1 min. To obtain dry powder samples, the obtained mixtures were freeze-dried (LGJ-10D, Beijing Science Instrument Co., Ltd., Beijing, China).

### Determination of physical properties of microcapsules

#### EE

EE refers to the mass ratio of encapsulated core material to total core material in microcapsule samples (24). The contents of surface flavonoids and total flavonoids in microcapsules were determined according to the method that Hu et al. (17) reported, and EE was calculated according to formula (1):


(1) $$\begin{array}{r} EE\;\left(\%\right)=\frac{TFC-SFC}{TFC}\;\times \;100 \end{array}$$

#### Moisture content (MC)

Water content is an important property of powder, which is closely related to its water activity, fluidity, stability, viscosity and microbial growth (25). Microcapsule samples (5 g) were dried in an oven at 70°C until constant weight (M_t_), the moisture content (MC) was calculated according to formula (2), where M_0_ is the initial weight of microcapsule sample:


(2) $$\begin{array}{r} MC\;\left(\%\right)=\frac{M_{0}-M_{t}}{M_{0}}\;\times \;100 \end{array}$$

#### Water solubility index (WSI)

The solubility was measured according to the method of Boyano Orozco et al. (26), with some modifications. Microcapsule samples were dispersed in distilled water, magnetically stirred at room temperature for 30 min, and immediately centrifuged at 5,000 rpm/min for 10 min. The supernatant was collected and transferred to a pre-weighed Petri dish for drying at 105°C. The water solubility index was calculated according to formula (3):


(3) $$\begin{array}{r} WSI\;\left(\%\right)\;=\;\frac{Weight\;of\;the\;sample\;in\;the\;supernatant}{Weight\;of\;the\;sample\;in\;the\;solution} \end{array}$$

#### Density and flowability

The bulk density (BD) and tap density (TD) were obtained by measuring the volume of 1 g microcapsule sample in a 10 ml measuring cylinder, and the fluidity of the microcapsule sample was assessed according to Carr's index (CI) (27). The calculation formula was as follows (4):


(4) $$\begin{array}{r} CI\;\left(\%\right)\;=\;\frac{Tapped\;density-Bulk\;density}{Tapped\;density} \end{array}$$

#### Color

Color difference meter (Xrite color i5, X-Rite of America, Michigan, USA) was used to determine the color of microcapsule samples, where L^*^ represents brightness (0 = black, 100 = white), a^*^ represents red index (–a^*^ is green, + a^*^ is red), b^*^ represents yellow index (–b^*^ is blue, + b^*^ is yellow), Chroma = (a^2^ + b^2^)^0.5^. Each sample was determined three times.

#### Determination of particle size

The preparation of the sample solution referred to Yu et al. (28), with some modifications. A certain amount of microcapsule powder was dispersed in ultrapure water, and placed in laser particle size analyzer (LA-960, HORIBA, Tokyo, Japan) to determine the particle size of microcapsule powder.

### Differential scanning calorimetry (DSC)

Differential scanning calorimetry can be used to analyze the thermal behavior of microcapsules and wall materials. Powder samples were determined by differential scanning calorimeter (Switzerland METTLER-TOLEDO, Zurich, Switzerland). Samples (3–5 mg) were placed in the crucible for DSC analysis at a nitrogen flow rate of 100 ml/min and a temperature rise rate of 10°C/min, ranging from 30°C to 300°C (29).

### Fourier transform infrared spectrometer (FT-IR)

FT-IR (VERTEX70, German BRUKER Company, Karlsruhe, German) was used to measure the chemical structure of raw materials and microcapsule powders at room temperature. The powder samples were mixed with the dried KBr at a ratio of 1:100 for measurement. The scanning range was 4,000–400 cm^−1^ and the spectral resolution was 4 cm^−1^ (30).

### Scanning electron microscopy (SEM)

The structural characteristics of microcapsules affect their physical and chemical properties and influence the ability of polymer wall material to protect core material (31). SEM (TM3030Plus, Hitachi High-Tech Corporation, Tokyo, Japan) was used to observe the surface micromorphology of microcapsules. The microcapsule powder was gently placed on the sample table and affixed with conductive tape, and the morphology of the microcapsule was photographed and observed in appropriate field of vision.

### Antioxidant activity

The DPPH radical scavenging ability of microcapsules was determined by ultraviolet spectrophotometry. Sample (200 μl) was added to 3.8 ml DPPH solution (6 × 10^−5^ mol/L), and kept in the dark for 30 min. The absorbance was measured at 517 nm, and the results were calculated based on vitamin C standard curve Equation 5, A_0_ and A_t_ were the absorbance of DPPH solution and the absorbance of sample mixed with DPPH solution, respectively.


(5) $$\begin{array}{r} DPPH\;radical\;scavenging\;efficiency\;\left(\%\right)=\frac{A_{0}-A_{t}}{A_{0}}\;\times \;100 \end{array}$$

## Results and discussion

### EE

In this study, three proteins were combined with MD to form wall materials at different protein/MD ratios (2:3, 3:7, 1:3), respectively. The effects of different protein/MD ratios on EE are shown in Table 1. The EE of microcapsules with different protein/MD ratios ranged from 93.44 to 98.27%, suggesting that these wall material compositions could realize satisfactory encapsulation. However, when the protein/MD ratio was 3:7, the EE value was higher than the other ratios. Clearly, the ratio of protein/MD as wall materials significantly influenced the EE of COF. When the ratio was 3:7, COF are better encapsulated in the wall material. The EE at the protein/MD ratio of 1:3 was lower than that at the ratio of 3:7. This could be due to the fact that the higher content of MD and the lower protein content was not favorable for enhancing EE. Therefore, the EE of COF was closely related to the wall material composition. The wall material composition induced different film-forming properties, which might contribute to the effect of encapsulating flavonoids. Similarly, Fredes et al. (32) prepared Maqui Juice microencapsulation using SPI and MD mixture as wall material, and EE of Maqui Juice by SD and FD could reach 92.5 and 93.0%, respectively. Norkaew et al. (33) also found that the EE of anthocyanins microcapsules with protein and MD as wall material almost reached 100%, which provided better protection for anthocyanins. Due to the higher EE at protein/MD ratio of 3:7, this ratio of 3:7 was selected to perform the following experiments.

**Table 1:** Effect of protein/MD ratios on EE of COF.

**Wall material composition**	**Protein/MD ratio**	**EE (%)**
WPI: MD	2:3	93.29 ± 0.41^e^
SPI: MD		93.44 ± 0.56^e^
GE: MD		93.63 ± 0.45^e^
WPI: MD	3:7	98.24 ± 0.48^a^
SPI: MD		97.82 ± 0.31^b^
GE: MD		96.32 ± 0.54^c^
WPI: MD	1:3	97.8 ± 0.44^b^
SPI: MD		96.3 ± 0.35^c^
GE: MD		95.4 ± 0.31^d^

Values are displayed as mean ± standard deviation. Different letters in the same column indicate significant differences (P < 0.05).

### Physical properties

Table 2 shows the results of MC, WSI, BD, TD, and CI of COF microencapsulation at the protein/MD ratio of 3:7. MC is an important index to measure the stability of powder products, which is related to drying efficiency, glass transition temperature, fluidity, storage stability, and so on (34). In addition, microcapsules in low moisture content are not prone to mildew and degradation, and can increase the stability of COF storage. In general, dry foods with MC between 4 and 10% have good storage stability (35). In our study, MC with different wall formulations ranged from 6.13 to 8.04%, which was conducive to maintain the stability of microcapsules during storage. GE-MD formulation had the highest MC (8.04%), while WPI-MD formulation had the lowest MC (6.13%) (Table 2), and protein types significantly affected the MC of microcapsules. The MC differences of microencapsulated sample might be related to differential affinity of proteins and water and different diffusion coefficients of water through the wall material.

**Table 2:** Physical properties of microcapsules.

**Encapsulating agent combination**	**MC (%)**	**WSI (%)**	**BD (g/ml)**	**TD (g/ml)**	**CI (%)**
WPI: MD (3:7)	6.13 ± 0.23^c^	90.10 ± 1.02^a^	0.28 ± 0.01^a^	0.42 ± 0.03^a^	33.33 ± 0.13^b^
SPI: MD (3:7)	7.22 ± 0.14^b^	89.20 ± 0.60^a^	0.25 ± 0.02^b^	0.39 ± 0.01^b^	35.90 ± 0.05^a^
GE: MD (3:7)	8.04 ± 0.17^a^	89.21 ± 0.20^a^	0.22 ± 0.02^c^	0.31 ± 0.01^c^	29.03 ± 0.31^c^

Values are displayed as mean ± standard deviation. Data with different superscript letters in the same column are significantly different (P < 0.05).

Water solubility index is a crucial factor to evaluate powder product as food ingredients, because those with poor solubility are not easy to further process and have low economic benefits. The results showed that all the microcapsules were in high solubility, which ranged from 89.2 to 90.1% (Table 2), but different wall material compositions had no significant discrepancy on the WSI of microcapsules (*P* < 0.05). Flavonoids were in low solubility, while the protein-polysaccharide mixture as wall material improved the solubility of COF. The high WSI of microcapsules were caused by that wall materials in high solubility and high content of MD was favorable for the solubility of core material (27).

The BD can reflect the gap size between powder particles, which is an essential parameter for assessing the texture. The BD of microcapsule powder is related to molecular weight of the wall material. The heavier material accommodates the less spaces between the particles, resulting in higher BD (36). As shown in Table 2, the BD of microcapsules prepared under different conditions ranged from 0.22 to 0.28 g/ml. The type of wall material has a significant effect on BD (*P* < 0.05). WPI combined with MD displayed a higher BD than the other two formulations, which may be caused by that WPI and MD combination was conducive to the formation of a more dense structure, resulting in a higher volume density. Higher BD indicates a lower amount of air in the powder void, which can prevent the oxidation of microcapsules so that the quality and preservation of microcapsule products can be guaranteed. For measuring TD, the space between particles is reduced due to the application of external force. The TD of the three types of microencapsulation ranged from 0.31 to 0.42 g/ml. The ratio of protein/MD had a significant effect on TD (*P* < 0.05). The microcapsule prepared by WPI-MD had higher TD and particles density, which indicated that a larger number of powder may be stored in smaller containers, which can reduce the air space of powder and oxidation of microcapsules to maintain the quality and stability of microcapsules (10). CI can be used to represent the free flow characteristics of powder. It can be seen from Table 2 that the CI is within the range of 30.67–35.9, indicating that these microcapsules have good fluidity. These results suggested that the combination of wall materials had a significant effect on the physical properties of microcapsules (*P* < 0.05).

### Color

Table 3 shows the color parameters of microcapsules with different wall material composition. The COF without encapsulation was red, and the freeze-dried powder prepared with WPI-MD and GE-MD was pink, but the powder prepared with SPI-MD was light pink. There were significant differences in L^*^, a^*^, b^*^ of COF microcapsules with different wall material (*P* < 0.05). Compared with the unencapsulated COF powder, the L^*^ of microcapsule powder was significantly increased, the a^*^ was significantly reduced, but the value of b^*^ had no change. The changes of L^*^ and a^*^ values were mainly due to the types of wall materials, which could mask the color of flavonoids. COF microcapsules powder prepared by WPI-MD and GE-MD were brighter than that prepared by SPI-MD, as the amount of COF exposed on their surface was less. Total color difference (ΔE) between control and microcapsule samples was also evaluated. There were significant differences between COF and microcapsules with different protein/MD combinations (*P* < 0.05). The ΔE order of microcapsules prepared by the three kinds of wall materials was as follows: GE-MD (27.11) > WPI-MD (25.33)>SPI-MD (23.57). High ΔE values indicated that the color change of microcapsules greatly increased, compared to unencapsulated COF.

**Table 3:** Color parameters of microcapsules.

**Wall material composition**	**Color**			
	L*	a*	b*	**ΔE**
COF	44.43 ± 0.33^a^	20.35 ± 0.7^a^	4.74 ± 0.43^b^	
WPI: MD (3:7)	67.84 ± 0.26^b^	10.75 ± 0.12^b^	3.48 ± 0.15^c^	25.33 ± 0.67^b^
SPI: MD (3:7)	64.20 ± 0.35^c^	7.53 ± 0.17^c^	5.30 ± 0.56^a^	23.57 ± 0.92^c^
GE: MD (3:7)	69.53 ± 0.67^a^	10.11 ± 0.53^b^	4.48 ± 0.13^b^	27.11 ±1.06^a^

Values are displayed as mean ± standard deviation. Different letters indicate significant (P < 0.05) differences between columns. The * symbol indicates the color attribute in table notes.

### Particle size

**Figure 1:**
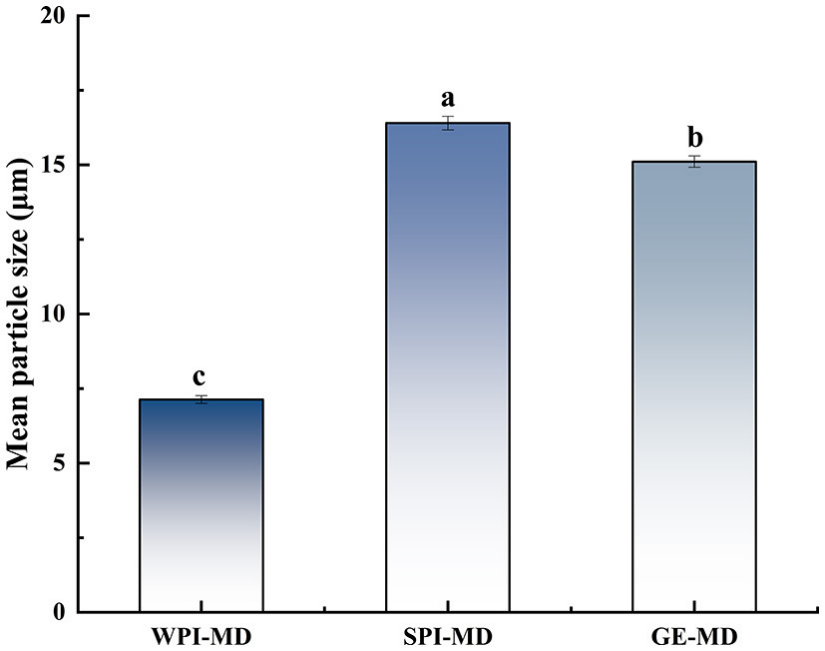
Mean particle size of microcapsules. Different superscript letters in the graph indicate significant differences between means (*P* < 0.05).

Particle size is an important parameter to evaluate the quality of microcapsule products. The smaller the particle size is, the easier the encapsulated bioactive compounds are to release (37). The average particle size of microcapsules with different wall materials formulations is shown in Figure 1, ranging from 7.17 to 16.4 μm. The particle size was significantly affected by the wall material (*P* <0.05). The microcapsule particle size was related to the molecular size of wall material. Different wall material mixed with COF possessed different structures and interaction force, which further influenced the particle size of microcapsules. Among the particles prepared with the three formulations, WPI-MD produced the smallest particles, which implied that WPI-MD-COF complex formed microcapsules with smaller structure. It can be seen that WPI-MD showed good encapsulating ability to form microcapsules. The particle size was also related to BD. The smaller the particle size is, the greater the BD is. It can be seen from Table 2 that the larger BD of microcapsules prepared with WPI-MD corresponded with the particle size.

### DSC analysis

**Figure 2:**
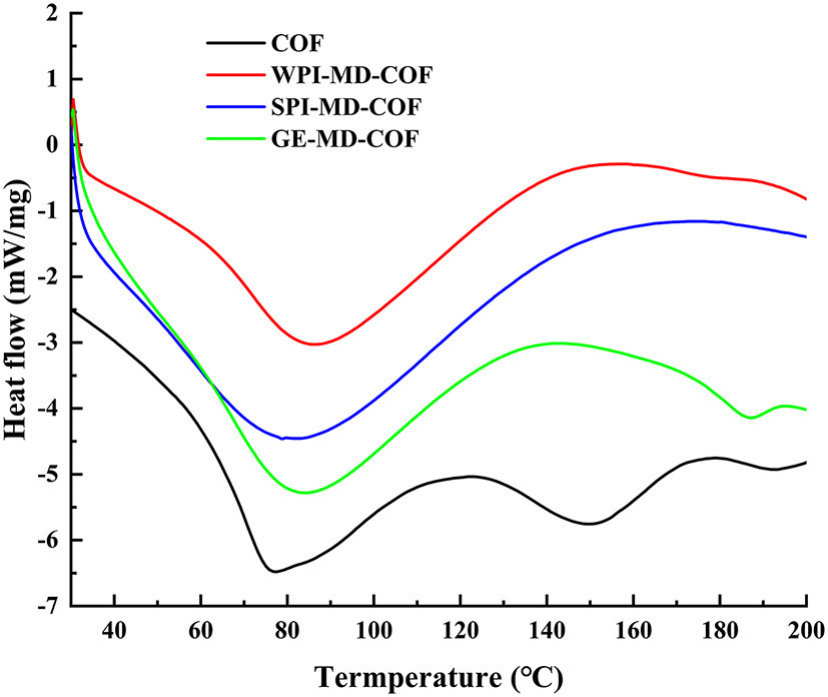
Differential scanning calorimetry (DSC) spectra of COF microcapsule particles of COF, WPI-MD-COF, SPI-MD-COF, and GE-MD-COF.

The thermal stability of microcapsule studied by DSC are shown in Figure 2. As can be observed, the thermal properties of microcapsules largely depended on the wall composition. COF powder showed endothermic peaks at 76°C and 150°C, which could be the denature and decomposition temperature of flavonoids, respectively. The COF loaded microcapsules showed the endothermic peaks (79.04°C−86.00°C) compared to single COF endothermic peak at 76°C, which implied that encapsulation changed the glass transition temperature of COF. This phenomenon suggests that the microencapsulation makes COF endothermic peaks shift to higher temperature, due to the interaction of COF and the wall material to increase the stability of the COF. In addition, the endothermic peak of COF at 150°C disappeared, which suggested that COF was embedded into the microcapsules. Microcapsules can maintain the stability of core material from heat treatment. Adsare et al. (16) also have obtained similar thermal behavior of curcumin microencapsulation using coconut milk whey and GA as wall material.

### FT-IR study

**Figure 3:**
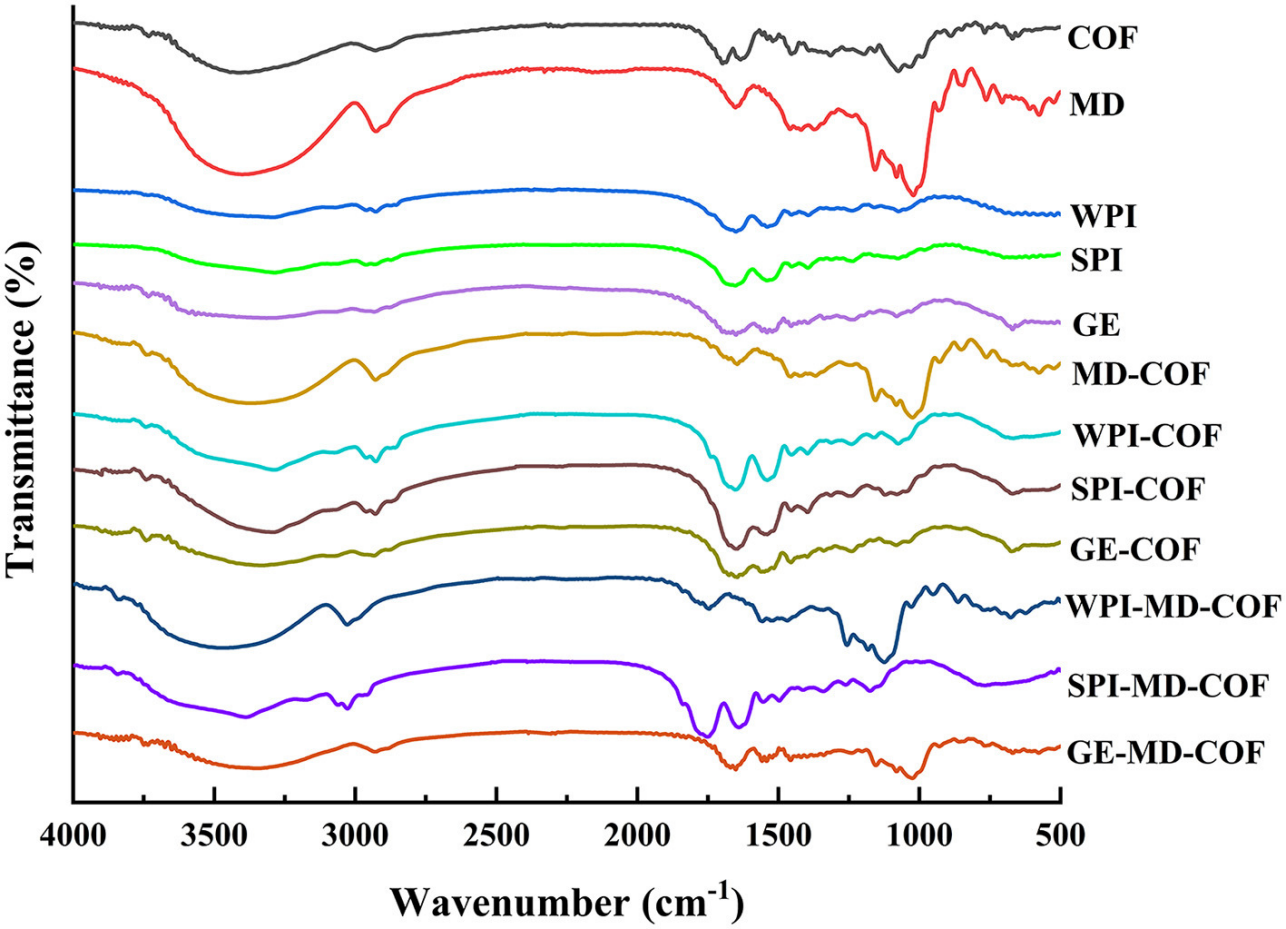
Infrared spectra of COF, MD, protein, protein-COF, and microcapsules.

The infrared spectrum can be used to analyze whether new functional groups and chemical bonds are introduced into flavonoids microcapsules. The infrared spectra of wall materials, core materials, and microcapsules are shown in Figure 3. The infrared spectrum of COF (Figure 3) showed a broad and strong peak formed by intermolecular hydrogen bonding at 3,408 cm^−1^, which proved existence of phenolic hydroxy groups, and the broad peak shape might be due to more hydroxyl groups on the flavonoid molecule (38); Meanwhile, asymmetric bending vibration peaks of –CH2 and –CH3 for COF were observed at 2,931 cm^−1^ and 1,316 cm^−1^, which proved more hydrogens on the saturated carbon. The peaks at 1,698 cm^−1^ and 1,634 cm^−1^ of COF were related to C = O and C = C stretching vibrational peaks, respectively. The infrared spectra of COF microcapsules prepared with different formulas were similar, and the specific peak of COF was retained, but the hydroxyl stretching vibration peak (3,288–3,383 cm^−1^) shifted to low wavenumbers, indicating that hydrogen bond formed between COF and wall material (39). Compared with individual MD and COF, the spectra of MD-COF mixture showed that the bands at 3,400 cm^−1^ of MD and 1,652 cm^−1^ of COF were shifted to lower wavenumbers, and the band at 1,081 cm^−1^ disappeared, which suggested there was interaction between MD and COF. After three kinds of proteins were individually mixed with COF, the hydroxyl stretching vibration peaks of COF were shifted to lower wavenumbers, C = O of WPI, SPI and GE moved from 1,651 cm^−1^, 1,652 cm^−1^ and 1,652 cm^−1^ to 1653 cm^−1^, 1,646 cm^−1^ and 1,646 cm^−1^, respectively. Besides, the bands of N-H bending at 1,395 cm^−1^, 1,456 cm^−1^, 1,396 cm^−1^ of WPI, SPI, and GE disappeared. The shifting in the amide region could confirm the interaction between COF and protein molecules with electrostatic forces (14).

Comparing the spectra of COF microcapsules prepared with combined wall materials, it was found that a strong C = O bond stretching vibration peak was formed in the three types of microcapsules at 1,652 cm^−1^, indicating that there was interaction between the wall material and COF (40). The spectra of microcapsules prepared by WPI-MD, SPI-MD, and GE-MD mixture indicated shifting of the hydroxyl peaks from 3,408 cm^−1^ of COF to 3,360, 3,361, and 3,358 cm^−1^, respectively, but the shift of the hydroxyl peaks was slight compared with their individual protein-COF mixture, due to the MD incorporation. Besides, during the formation of flavonoid microcapsules, the peak shifted from 1,453 cm^−1^ of COF to 1,454 (WPI-MD-COF), 1,455 (SPI-MD-COF) and 1,457 (GE-MD-COF) cm^−1^and the peak width of microcapsules narrowed. Compared with the COF spectra, the characteristic peaks of the three microcapsules at 1,075 cm^−1^ and 1,315 cm^−1^ disappeared, indicating that the COF was encapsulated in the microcapsules. The FT-TR results also supported the differences on DSC spectra of COF and COF microcapsules, which confirmed the interaction between the wall material and the core material, providing the evidences on successfully encapsulating COF.

### Microcapsule morphology

**Figure 4:**
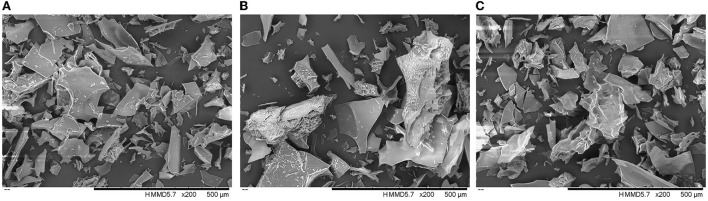
Scanning electron microscopy (SEM) of WPI-MD-COF **(A)**, SPI-MD-COF **(B)**, and GE-MD-COF **(C)** microcapsules.

The morphology of microcapsule powder with different formulations is shown in Figure 4. The surface of microcapsules presented flake structures of different sizes, which was a common feature of FD particles. The microcapsules showed changes in surface microstructure due to the different wall composition (41). Microcapsules prepared with WPI-MD and GE-MD formulations showed glassy structures and smooth surfaces, whereas microcapsules prepared with SPI-MD mixture had irregular shapes and porous surfaces. This was due to the formation of ice crystals during the pre-freezing process of the microencapsulated mixture, which further underwent the sublimation of water vapor and the formation of irregular porous pores. The formed ice crystals at pre-freezing stage destroyed the original structure of wall materials, and caused solute polymerization, contributing to the structure change of different wall materials under MFD condition. The water sublimation rate of COF microcapsules during MFD process was also affected by wall materials, which led to the morphology differences among different COF microcapsules. These changes destroyed the original structure of the material and changed the physical properties of microcapsules (42, 43). Similar glassy results were observed on the morphology of anthocyanin encapsulated by MD and GA using freeze-drying techniques (44).

### Antioxidant activity

**Figure 5:**
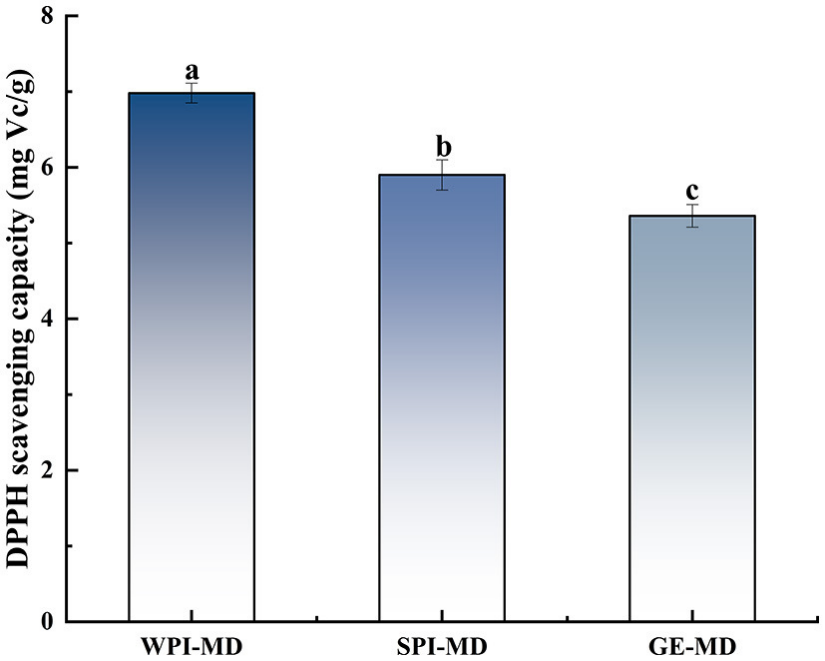
The DPPH free radical scavenging ability of microcapsules. Different superscript letters in the graph indicate significant differences between means (*P* < 0.05).

The DPPH radical scavenging ability of microcapsules with different wall material formulations is illustrated in Figure 5. The results revealed that the EE with different wall materials showed different antioxidant ability, and the DPPH radical scavenging ability of COF microcapsules prepared with three protein-polysaccharide mixtures ranged from 5.36 mg Vc/g to 6.98 mg Vc/g. Results showed that the mixture of WPI-MD (3:7) had the highest DPPH free radical scavenging ability, and the mixture of GE-MD (3:7) had the lowest DPPH radical scavenging ability. These results were consistent with the same trend in EE, suggesting that COF in microcapsules played a main role in exhibiting DPPH radical scavenging ability. Moser et al. (45) also proved that anthocyanins encapsulated by WPI-MD wall combinations exerted high DPPH radical scavenging ability (8.6 mmol Trolox/100 g).

## Conclusions

In this study, protein (WPI, SPI, and GE) and MD was both used as wall material to prepare COF microcapsules by FD. The effect of the wall material formulation on characteristics of COF microcapsules was explored. The EE of COF microcapsules at protein/MD ratio of 3:7 was higher than the other ratios. The variety of proteins significantly affected the characteristics of microcapsules. Compared with free COF, microencapsulation can improve thermal stability of COF. FT-IR showed that flavonoids were successfully encapsulated in the wall materials. The microstructure of all the microcapsules exhibited irregular lamellar structure. COF microcapsules prepared by WPI-MD showed higher DPPH scavenging ability. Among the three wall material formulations, WPI-MD mixture displayed more advantages on its application into food industry. This study provided more evidences for developing COF microcapsules as functional food. In future, more researches on investigating controlled release of COF microcapsules in specific food should be further carried out.

## Data availability statement

The original contributions presented in the study are included in the article/supplementary material, further inquiries can be directed to the corresponding author/s.

## Author contributions

XD, AR, and WC: conceptualization. XD, GR, and LL: methodology. MZ: investigation and writing—original draft preparation. MZ, XR, and YA: software. MZ, YA, and ZW: data curation. XD and WC: writing—review and editing. GR, JC, and BB: supervision. XD, AR, and GR: funding acquisition. All authors contributed to the article and approved the submitted manuscript.

## Funding

We acknowledge the financial support from National Natural Science Foundation of China (Contract Nos. 32172352 and 31972207), National Natural Science Foundation of China Regional Fund Project (Contract No. 32160573), Key Scientific Research Projects of Institutions of Higher Learning of Henan (Contract No. 22A550005), Key Science and Technology Program of Henan Province (Contract No. 222102520011), Young Teacher Funding Program of the Henan Higher School (Contract No. 2020GGJS072), Guangxi Natural Science Foundation of China (Contract No. 2020GXNSFAA259093), and Special Program for the Introduction of Foreign Intelligence in Henan Province (Foreign Experts Project) (Contract No. HNGD2021040), all of which enabled us to carry out this study.

## Conflict of interest

The authors declare that the research was conducted in the absence of any commercial or financial relationships that could be construed as a potential conflict of interest.

## Publisher's note

All claims expressed in this article are solely those of the authors and do not necessarily represent those of their affiliated organizations, or those of the publisher, the editors and the reviewers. Any product that may be evaluated in this article, or claim that may be made by its manufacturer, is not guaranteed or endorsed by the publisher.
